# Supplementary material for: Development of a computer-aided design software for the quantitative evaluation of aesthetic damage
Source: PLoS One. 2019 Dec 18;14(12):e0226322. doi: 10.1371/journal.pone.0226322 (PMC6919621; doi:10.1371/journal.pone.0226322)
Supplement: S1 Code site — 1. (ZIP) [file pone.0226322.s001.zip › [EESC_jr][Projeto]Relatorio_07_21/reports/templates_zip/gentelella-master.zip!/gentelella-master/production/glyphicons.html]

Gentellela Alela! | 


Gentellela Alela!

Welcome,

## John Doe

  


### General

- Home 
  - Dashboard
  - Dashboard2
  - Dashboard3
- Forms 
  - General Form
  - Advanced Components
  - Form Validation
  - Form Wizard
  - Form Upload
  - Form Buttons
- UI Elements 
  - General Elements
  - Media Gallery
  - Typography
  - Icons
  - Glyphicons
  - Widgets
  - Invoice
  - Inbox
  - Calendar
- Tables 
  - Tables
  - Table Dynamic
- Data Presentation 
  - Chart JS
  - Chart JS2
  - Moris JS
  - ECharts
  - Other Charts
- Layouts 
  - Fixed Sidebar
  - Fixed Footer

### Live On

- Additional Pages 
  - E-commerce
  - Projects
  - Project Detail
  - Contacts
  - Profile
- Extras 
  - 403 Error
  - 404 Error
  - 500 Error
  - Plain Page
  - Login Page
  - Pricing Tables
- Multilevel Menu 
  - Level One- Level One
      - Level Two
      - Level Two
      - Level Two
    - Level One
- Landing Page Coming Soon

- John Doe
  - Profile
  - 50%
    Settings
  - Help
  - Log Out
- 6
  - John Smith
    3 mins ago

    Film festivals used to be do-or-die moments for movie makers. They were where...
  - John Smith
    3 mins ago

    Film festivals used to be do-or-die moments for movie makers. They were where...
  - John Smith
    3 mins ago

    Film festivals used to be do-or-die moments for movie makers. They were where...
  - John Smith
    3 mins ago

    Film festivals used to be do-or-die moments for movie makers. They were where...
  - **See All Alerts**


### Glyphicons Icons

Go!

## Glyphicons different icon design elements

- - Settings 1
  - Settings 2

# Glyphicons

## Available glyphs

Includes 260 glyphs in font format from the Glyphicon Halflings set. Glyphicons Halflings are normally not available for free, but their creator has made them available for Bootstrap free of cost.
As a thank you, we only ask that you include a link back to Glyphicons whenever possible.

- glyphicon glyphicon-asterisk
- glyphicon glyphicon-plus
- glyphicon glyphicon-euro
- glyphicon glyphicon-eur
- glyphicon glyphicon-minus
- glyphicon glyphicon-cloud
- glyphicon glyphicon-envelope
- glyphicon glyphicon-pencil
- glyphicon glyphicon-glass
- glyphicon glyphicon-music
- glyphicon glyphicon-search
- glyphicon glyphicon-heart
- glyphicon glyphicon-star
- glyphicon glyphicon-star-empty
- glyphicon glyphicon-user
- glyphicon glyphicon-film
- glyphicon glyphicon-th-large
- glyphicon glyphicon-th
- glyphicon glyphicon-th-list
- glyphicon glyphicon-ok
- glyphicon glyphicon-remove
- glyphicon glyphicon-zoom-in
- glyphicon glyphicon-zoom-out
- glyphicon glyphicon-off
- glyphicon glyphicon-signal
- glyphicon glyphicon-cog
- glyphicon glyphicon-trash
- glyphicon glyphicon-home
- glyphicon glyphicon-file
- glyphicon glyphicon-time
- glyphicon glyphicon-road
- glyphicon glyphicon-download-alt
- glyphicon glyphicon-download
- glyphicon glyphicon-upload
- glyphicon glyphicon-inbox
- glyphicon glyphicon-play-circle
- glyphicon glyphicon-repeat
- glyphicon glyphicon-refresh
- glyphicon glyphicon-list-alt
- glyphicon glyphicon-lock
- glyphicon glyphicon-flag
- glyphicon glyphicon-headphones
- glyphicon glyphicon-volume-off
- glyphicon glyphicon-volume-down
- glyphicon glyphicon-volume-up
- glyphicon glyphicon-qrcode
- glyphicon glyphicon-barcode
- glyphicon glyphicon-tag
- glyphicon glyphicon-tags
- glyphicon glyphicon-book
- glyphicon glyphicon-bookmark
- glyphicon glyphicon-print
- glyphicon glyphicon-camera
- glyphicon glyphicon-font
- glyphicon glyphicon-bold
- glyphicon glyphicon-italic
- glyphicon glyphicon-text-height
- glyphicon glyphicon-text-width
- glyphicon glyphicon-align-left
- glyphicon glyphicon-align-center
- glyphicon glyphicon-align-right
- glyphicon glyphicon-align-justify
- glyphicon glyphicon-list
- glyphicon glyphicon-indent-left
- glyphicon glyphicon-indent-right
- glyphicon glyphicon-facetime-video
- glyphicon glyphicon-picture
- glyphicon glyphicon-map-marker
- glyphicon glyphicon-adjust
- glyphicon glyphicon-tint
- glyphicon glyphicon-edit
- glyphicon glyphicon-share
- glyphicon glyphicon-check
- glyphicon glyphicon-move
- glyphicon glyphicon-step-backward
- glyphicon glyphicon-fast-backward
- glyphicon glyphicon-backward
- glyphicon glyphicon-play
- glyphicon glyphicon-pause
- glyphicon glyphicon-stop
- glyphicon glyphicon-forward
- glyphicon glyphicon-fast-forward
- glyphicon glyphicon-step-forward
- glyphicon glyphicon-eject
- glyphicon glyphicon-chevron-left
- glyphicon glyphicon-chevron-right
- glyphicon glyphicon-plus-sign
- glyphicon glyphicon-minus-sign
- glyphicon glyphicon-remove-sign
- glyphicon glyphicon-ok-sign
- glyphicon glyphicon-question-sign
- glyphicon glyphicon-info-sign
- glyphicon glyphicon-screenshot
- glyphicon glyphicon-remove-circle
- glyphicon glyphicon-ok-circle
- glyphicon glyphicon-ban-circle
- glyphicon glyphicon-arrow-left
- glyphicon glyphicon-arrow-right
- glyphicon glyphicon-arrow-up
- glyphicon glyphicon-arrow-down
- glyphicon glyphicon-share-alt
- glyphicon glyphicon-resize-full
- glyphicon glyphicon-resize-small
- glyphicon glyphicon-exclamation-sign
- glyphicon glyphicon-gift
- glyphicon glyphicon-leaf
- glyphicon glyphicon-fire
- glyphicon glyphicon-eye-open
- glyphicon glyphicon-eye-close
- glyphicon glyphicon-warning-sign
- glyphicon glyphicon-plane
- glyphicon glyphicon-calendar
- glyphicon glyphicon-random
- glyphicon glyphicon-comment
- glyphicon glyphicon-magnet
- glyphicon glyphicon-chevron-up
- glyphicon glyphicon-chevron-down
- glyphicon glyphicon-retweet
- glyphicon glyphicon-shopping-cart
- glyphicon glyphicon-folder-close
- glyphicon glyphicon-folder-open
- glyphicon glyphicon-resize-vertical
- glyphicon glyphicon-resize-horizontal
- glyphicon glyphicon-hdd
- glyphicon glyphicon-bullhorn
- glyphicon glyphicon-bell
- glyphicon glyphicon-certificate
- glyphicon glyphicon-thumbs-up
- glyphicon glyphicon-thumbs-down
- glyphicon glyphicon-hand-right
- glyphicon glyphicon-hand-left
- glyphicon glyphicon-hand-up
- glyphicon glyphicon-hand-down
- glyphicon glyphicon-circle-arrow-right
- glyphicon glyphicon-circle-arrow-left
- glyphicon glyphicon-circle-arrow-up
- glyphicon glyphicon-circle-arrow-down
- glyphicon glyphicon-globe
- glyphicon glyphicon-wrench
- glyphicon glyphicon-tasks
- glyphicon glyphicon-filter
- glyphicon glyphicon-briefcase
- glyphicon glyphicon-fullscreen
- glyphicon glyphicon-dashboard
- glyphicon glyphicon-paperclip
- glyphicon glyphicon-heart-empty
- glyphicon glyphicon-link
- glyphicon glyphicon-phone
- glyphicon glyphicon-pushpin
- glyphicon glyphicon-usd
- glyphicon glyphicon-gbp
- glyphicon glyphicon-sort
- glyphicon glyphicon-sort-by-alphabet
- glyphicon glyphicon-sort-by-alphabet-alt
- glyphicon glyphicon-sort-by-order
- glyphicon glyphicon-sort-by-order-alt
- glyphicon glyphicon-sort-by-attributes
- glyphicon glyphicon-sort-by-attributes-alt
- glyphicon glyphicon-unchecked
- glyphicon glyphicon-expand
- glyphicon glyphicon-collapse-down
- glyphicon glyphicon-collapse-up
- glyphicon glyphicon-log-in
- glyphicon glyphicon-flash
- glyphicon glyphicon-log-out
- glyphicon glyphicon-new-window
- glyphicon glyphicon-record
- glyphicon glyphicon-save
- glyphicon glyphicon-open
- glyphicon glyphicon-saved
- glyphicon glyphicon-import
- glyphicon glyphicon-export
- glyphicon glyphicon-send
- glyphicon glyphicon-floppy-disk
- glyphicon glyphicon-floppy-saved
- glyphicon glyphicon-floppy-remove
- glyphicon glyphicon-floppy-save
- glyphicon glyphicon-floppy-open
- glyphicon glyphicon-credit-card
- glyphicon glyphicon-transfer
- glyphicon glyphicon-cutlery
- glyphicon glyphicon-header
- glyphicon glyphicon-compressed
- glyphicon glyphicon-earphone
- glyphicon glyphicon-phone-alt
- glyphicon glyphicon-tower
- glyphicon glyphicon-stats
- glyphicon glyphicon-sd-video
- glyphicon glyphicon-hd-video
- glyphicon glyphicon-subtitles
- glyphicon glyphicon-sound-stereo
- glyphicon glyphicon-sound-dolby
- glyphicon glyphicon-sound-5-1
- glyphicon glyphicon-sound-6-1
- glyphicon glyphicon-sound-7-1
- glyphicon glyphicon-copyright-mark
- glyphicon glyphicon-registration-mark
- glyphicon glyphicon-cloud-download
- glyphicon glyphicon-cloud-upload
- glyphicon glyphicon-tree-conifer
- glyphicon glyphicon-tree-deciduous
- glyphicon glyphicon-cd
- glyphicon glyphicon-save-file
- glyphicon glyphicon-open-file
- glyphicon glyphicon-level-up
- glyphicon glyphicon-copy
- glyphicon glyphicon-paste
- glyphicon glyphicon-alert
- glyphicon glyphicon-equalizer
- glyphicon glyphicon-king
- glyphicon glyphicon-queen
- glyphicon glyphicon-pawn
- glyphicon glyphicon-bishop
- glyphicon glyphicon-knight
- glyphicon glyphicon-baby-formula
- glyphicon glyphicon-tent
- glyphicon glyphicon-blackboard
- glyphicon glyphicon-bed
- glyphicon glyphicon-apple
- glyphicon glyphicon-erase
- glyphicon glyphicon-hourglass
- glyphicon glyphicon-lamp
- glyphicon glyphicon-duplicate
- glyphicon glyphicon-piggy-bank
- glyphicon glyphicon-scissors
- glyphicon glyphicon-bitcoin
- glyphicon glyphicon-yen
- glyphicon glyphicon-ruble
- glyphicon glyphicon-scale
- glyphicon glyphicon-ice-lolly
- glyphicon glyphicon-ice-lolly-tasted
- glyphicon glyphicon-education
- glyphicon glyphicon-option-horizontal
- glyphicon glyphicon-option-vertical
- glyphicon glyphicon-menu-hamburger
- glyphicon glyphicon-modal-window
- glyphicon glyphicon-oil
- glyphicon glyphicon-grain
- glyphicon glyphicon-sunglasses
- glyphicon glyphicon-text-size
- glyphicon glyphicon-text-color
- glyphicon glyphicon-text-background
- glyphicon glyphicon-object-align-top
- glyphicon glyphicon-object-align-bottom
- glyphicon glyphicon-object-align-horizontal
- glyphicon glyphicon-object-align-left
- glyphicon glyphicon-object-align-vertical
- glyphicon glyphicon-object-align-right
- glyphicon glyphicon-triangle-right
- glyphicon glyphicon-triangle-left
- glyphicon glyphicon-triangle-bottom
- glyphicon glyphicon-triangle-top
- glyphicon glyphicon-console
- glyphicon glyphicon-superscript
- glyphicon glyphicon-subscript
- glyphicon glyphicon-menu-left
- glyphicon glyphicon-menu-right
- glyphicon glyphicon-menu-down
- glyphicon glyphicon-menu-up

## How to use

For performance reasons, all icons require a base class and individual icon class. To use, place the following code just about anywhere. Be sure to leave a space between the icon and text for proper padding.

#### Don't mix with other components

Icon classes cannot be directly combined with other components. They should not be used along with other classes on the same element. Instead, add a nested `<span>` and apply the icon classes to the `<span>`.

#### Only for use on empty elements

Icon classes should only be used on elements that contain no text content and have no child elements.

#### Changing the icon font location

Bootstrap assumes icon font files will be located in the `../fonts/` directory, relative to the compiled CSS files. Moving or renaming those font files means updating the CSS in one of three ways:

- Change the `@icon-font-path` and/or `@icon-font-name` variables in the source Less files.
- Utilize the relative URLs option provided by the Less compiler.
- Change the `url()` paths in the compiled CSS.

Use whatever option best suits your specific development setup.

#### Accessible icons

Modern versions of assistive technologies will announce CSS generated content, as well as specific Unicode characters. To avoid unintended and confusing output in screen readers (particularly when icons are used purely for decoration),
we hide them with the `aria-hidden="true"` attribute.

If you're using an icon to convey meaning (rather than only as a decorative element), ensure that this meaning is also conveyed to assistive technologies – for instance, include additional content, visually hidden with the `.sr-only` class.

If you're creating controls with no other text (such as a `<button>` that only contains an icon), you should always provide alternative content to identify the purpose of the control, so that it will make sense to
users of assistive technologies. In this case, you could add an `aria-label` attribute on the control itself.

Copy

```
<span class="glyphicon glyphicon-search" aria-hidden="true"></span>
```

## Examples

Use them in buttons, button groups for a toolbar, navigation, or prepended form inputs.

Star
 Star
 Star
 Star

Copy

```
<button type="button" class="btn btn-default" aria-label="Left Align">
  <span class="glyphicon glyphicon-align-left" aria-hidden="true"></span>
</button>

<button type="button" class="btn btn-default btn-lg">
  <span class="glyphicon glyphicon-star" aria-hidden="true"></span> Star
</button>
```

An icon used in an alert to convey that it's an error message, with additional `.sr-only` text to convey this hint to users of assistive technologies.

Error: Enter a valid email address

Copy

```
<div class="alert alert-danger" role="alert">
  <span class="glyphicon glyphicon-exclamation-sign" aria-hidden="true"></span>
  <span class="sr-only">Error:</span>
  Enter a valid email address
</div>
```


Gentelella - Bootstrap Admin Template by Colorlib
